# Parent knowledge of and attitudes towards youth sport-related concussion and associations with child and parent factors

**DOI:** 10.2217/cnc-2021-0008

**Published:** 2022-04-07

**Authors:** Samantha D Roberts, Phillip Schatz, Johna Register-Mihalik, Magdalena Wojtowicz

**Affiliations:** 1 Department of Psychology, York University, Toronto, ON, M3J 1P3, Canada; 2 Department of Psychology, Saint Joseph's University, Philadelphia, PA 19131, USA; 3 Department of Exercise & Sport Science, University of North Carolina, Chapel Hill, NC 27514, USA

**Keywords:** attitudes, parent knowledge, perceived knowledge, sport-related concussion, youth sport

## Abstract

**Aim:**

This cross-sectional study aimed to better understand parental knowledge and attitudes regarding pediatric sport-related concussions, and association with parent/child biopsychosocial factors.

**Methods:**

A community sample of ninety families (n = 140 children) were included. Parental concussion knowledge and attitudes, concussion history, sport participation and social risk status score (SRS) were collected.

**Results:**

Parents scored an average of 76% accuracy on factual concussion knowledge, with 74% confidence in responses. Parents endorsed a favorable attitude toward concussion reporting and management. Low SRS had higher perceived accuracy of knowledge than medium or high SRS (p = 0.003). SRS influenced over-and-underestimations of factual knowledge (p = .04). Age at first sport and sport contact level influenced factual and perceived concussion knowledge.

**Conclusion:**

These findings identify common gaps in concussion knowledge in parents.

Youth sport participation is associated with numerous physical, psychological and sociological benefits [1]. Children who engage in sports are less likely to develop heart disease, obesity, diabetes, stroke and depression later in life [2–4]. Furthermore, sport participation has been linked to enhanced emotional regulation, social skills, athletic skills, building peer relationships and heightened self-esteem [5–10]. Despite these many benefits, there is growing concern about potential adverse aspects of sport participation, including the risk of sustaining one or multiple concussions. Although most youth athletes who sustain a sport-related concussion fully recover from their symptoms [11], a subset of youth experience more adverse outcomes such as cognitive, behavioural and socio-emotional difficulties [12]. For youth athletes, parents play a major role in identifying concussive symptoms, managing symptoms, at-home recovery and making initial decisions about return to play [13]. In this regard, the scope and accuracy of parental concussion knowledge can influence attitudes toward sport-related concussion and, in-turn, influence parental decisions about keeping youth in sport.

Current research has focused primarily on understanding the knowledge and attitudes toward sport-related concussion among adolescent and young adult athletes, and coaches [14–16]. The limited research examining parental knowledge of youth concussion has found that although the majority of parents have heard about concussion, only one-quarter of parents reported a basic understanding of concussion symptoms and diagnosis criteria [17]. Further, researchers in the USA reported that the average parent concussion knowledge score was 73%, with scores ranging from 24 to 96% (higher knowledge scores indicate more accurate knowledge about concussions), and the average parental concussion attitude score was 84%, representing a positive attitude toward concussion reporting and management [13,18]. Interestingly, research on parent and child concussion history and sport participation as moderators of parental knowledge or attitude scores remains equivocal [13]. A more comprehensive understanding of such parental and child factors may help further refine education platforms and identify families that may benefit from targeted concussion management initiatives.

Parental knowledge and attitudes about concussion reporting, management and treatment may be moderated by several family and child factors, such as socioeconomic status and education [19,20], age of parent and child [17], and sport-contact level of the child. Social risk status can be measured by quantifying six aspects of social status: family structure, education and occupation of primary caregiver, employment of primary income earner, maternal age at birth, and language spoken in the home [21,22]. The factors that are encompassed in family social risk status individually have an impact on the level of youth sport participation, as well as on parental confidence and attitudes [19]. Children who have higher socioeconomic status (SES) are likely to engage in more hours of physical activity and have increased participation in sports compared with children with lower SES [23–25]. Parents with higher SES are most likely to have the financial means and free time to provide their children access to sports compared with parents with lower SES [24,26]. Factors such as parental education may influence the level of understanding that parents have on the positive health outcomes of youth sport participation [24,27,28]. Parents with higher education may, in turn, be more supportive and encouraging of athletic participation [19,28]. The level of parental education may impact the degree of understanding adverse facets of sport participation, such as concussions [13]. Last, there is strong evidence for the negative impact that stress associated with low SES has on parent attitudes and confidence in general [29,30]. There is some evidence to suggest that lower parental socioeconomic status may adversely influence the recognition of concussion signs, symptoms, and knowledge of return-to-play protocols [20]. However, understanding how social risk status influences parental knowledge and attitudes of pediatric sport-related concussions remains elusive.

The few Canadian studies that examined concussion knowledge used mixed samples of healthcare providers, coaches, athletes, teachers and parents, finding that a significant amount of Canadians have a basic understanding of what a concussion is but lack the knowledge about details of a concussion [31,32]. They identified that virtually all Canadians surveyed believe concussion is an important health issue (97%); however, a limited amount knew where to get concussion information (51%), how to recognize signs and symptoms (46%) and what to do if someone sustains a concussion (40%) [32]. In Canada, there is a limited understanding of the level of knowledge and associated attitudes about pediatric sport-related concussion reporting and management among parents. Furthermore, there is a paucity of research that examines factors that may influence parental knowledge and attitudes of concussion reporting and management.

There were two objectives for the current study. The first objective was to understand the relationship between parental knowledge of and attitudes toward youth concussion and comprehensive parental factors (i.e., social risk status, age of parent, parent concussion history, sex of the informant, number of children). The second objective was to examine parent-reported child factors (e.g., child concussion history, sport contact level, child's age, child's age at first sport) that may influence parental knowledge of and attitudes toward youth concussion.

## Methods

### Participants

A community-based sample of parents/primary caregivers were recruited from the greater Toronto area (GTA). Parents were eligible to participate if they were: >18 years of age, able to read and speak English at a grade 7 literacy level (self-declared) and parent/primary caregiver of children between the ages of 6 and 14 years, regardless of sport participation. Parents who had multiple children between 6 and 14 years were asked to complete child-related questions for each child. There were 105 families enrolled in the study; 15 families were removed from the final sample due to poor completion rates (<40% of study questionnaires completed). The final sample was comprised of 90 parents (n = 140 children) with 87% of the sample having 100% completion rate.

### Procedure

Participants were recruited from athletic organizations, libraries, community centers and a local festival in the GTA. In order to maximize recruitment, participants were recruited using an in-person approach which provided access to the online study questionnaire, or a link to complete the questionnaire at a later time. Potential parent participants were approached by one of the research team members who introduced and explained the research study, as well as eligibility criteria. If the eligible parent expressed interest, the research team member obtained verbal consent to contact the parent. Eligible parents were provided with the study questionnaire on an Apple iPad by a study team member to complete in-person via Qualtrics (online secure/anonymous survey platform) or the study link to complete at home where they completed the online written consent form and the study questionnaire. The study questionnaire was approximately 10–15 min in length and was developed at a Grade 5 literacy level.

### Measures

#### Demographic, sport & concussion information

Parent and child demographic information was collected about both the parent and child. Parents answered questions about parental and child concussion history and child sport participation history (i.e., past and current sports, child's level of sport) and current parents' sport participation. Child sport participation included a variety of sports played within the community, school, or organized sport teams (Table 1), and they were categorized based on the level of contact (i.e. collision, contact, or limited/no contact, based on American Academy of Pediatrics guidelines [33]) for analysis purposes. For those enrolled in multiple sports, they were coded based on the highest level of contact of the sports they currently participate in.

**Table 1.** Participant descriptives.

Parent factors (n = 90)	n (%) or mean (standard deviation)
Parent or caregiver participating	
Mother	66 (73%)
Father	21 (23%)
Both parents/caregiver	4 (4%)
Number of children in the family	
One child	47 (52%)
Two children	36 (40%)
Three children	7 (8%)
Social risk status score^†^	
Low risk (0)	62 (69%)
Medium risk (1)	13 (14%)
High risk (2+)	14 (15%)
Parent age	41 years 8 months (6 years 1 month)
Parents with a concussion history	28 (31%)
Range of concussions	1–5
Factual knowledge score	21.29 (2.44)
Perceived knowledge score	15.66 (2.66)
Attitude score	48.45 (4.46)

Child factors (n = 140)	
Child age	9 years 5 months (2 years 10 months)
Child age when they first began sports	3 years 10 months (1 years 3 months)
Children with a concussion history	23 (16%)
Range of concussions	1–3
Child sport contact level^‡^	
Collision	49 (37%)
Contact	42 (31%)
Limited/no contact	41 (31%)

† Six aspects of the social risk status score that are summed for total score: maternal age at birth (2 – <18 years old, 1 – 18–21 years old, or 0 – >21 years old), language spoken at home (2 – no English, 1 – some English, 0 – only English), education of primary income earner (2 – less than high school diploma, 1 – high school, 0 – college, university, or graduate studies), employment status of primary income earner (2 – unemployed, 1 – part time, 0 – full time), occupation or primary income earner (2 – unskilled, 1 – semi–skilled, 0 – skilled/professional), and family living structure (2 – single caregiver, 1 – separated parents with dual custody or cared for by other intact family, 0 – two caregivers). One parent did not provide information on social risk factors.

‡ Collision included football, rugby, hockey and lacrosse; Contact included flag football, basketball, dodgeball, cheerleading, wrestling, and soccer; and Limited/no contact included baseball, volleyball, dance, equestrian sport, gymnastics, swimming, track and field and water polo.

#### Social risk score

Using specific demographic information, social risk was computed and categorized based on a previously validated rank system [21,22]. The total social risk score is a composite score comprised of six aspects of social status scored as low social risk (score of zero), medium social risk (score of one), or high social risk (score of two) based on follow-up studies of other medically at-risk children [21]: maternal age at birth (2 – less than 18 years old, 1 – 18–21 years old, or 0 – more than 21 years old), language spoken at home (2 – no English, 1 – some English, 0 – only English), education of primary income earner (2 – less than high school diploma, 1 – high school, 0 – college, university, or graduate studies), employment status of primary income earner (2 – unemployed, 1 – part time, 0 – full time), occupation or primary income earner (2 – unskilled, 1 – semi-skilled, 0 – skilled/professional), and family living structure (2 – single caregiver, 1 – separated parents with dual custody or cared for by other intact family, 0 – two caregivers). The total social risk score (SRS) was calculated by summing the scores for the six aspects (Table 1). Each family was categorized as low social risk (total SRS = 0), medium social risk (total SRS = 1), or high social risk (total SRS = 2+).

#### Rosenbaum concussion knowledge & attitudes survey

The Rosenbaum concussion knowledge and attitudes survey (RoCKAS), is a 55 item, 5 section questionnaire examining concussion knowledge [34]. Sections 1, 2 and 5 from the RoCKAS were used to assess parental *factual* knowledge of concussions. A total *factual* concussion knowledge score was calculated by summing all of the points obtained in the three sections, with a higher score demonstrating more *factual* knowledge about concussions (total scores range from 12 to 25). This measure displays moderate psychometric properties (intraclass correlation coefficient of 0.67) [34].

#### Perceived knowledge

Participants were asked to rate how confident they feel about their answers to the factual knowledge sections from the RoCKAS on a 7-point Likert-scale (range 1–7). A total *perceived* knowledge score was calculated by summing their responses (total score range from 3 to 21). Higher scores represent more *perceived* knowledge about concussions.

#### Parent concussion survey

The parent concussion survey (PCS) is a questionnaire that assesses knowledge of and attitudes toward concussions [35]. For the purposes of this study, only parental attitudes questions were used from the PCS. Participants' attitudes about concussion were assessed by the nine 7-point Likert-scale questions, which asked about attitudes toward concussion management and return to play decisions. A total attitude score was calculated by summing the responses (total score range of 9–63). A higher score represents a more favorable attitude toward reporting concussions, importance of reporting a possible concussion and an overall positive view of concussion management [18]. This measure demonstrates good psychometric properties, specifically internal consistency has been found to be acceptable for the attitude construct, with Cronbach alpha beyond 0.70 [35].

### Statistical analysis

Summary descriptive statistics were calculated to describe the sample characteristics, pattern of knowledge about concussions and attitudes toward concussion reporting and treatment. Discrepancy scores were calculated between the parental factual and perceived knowledge to examine over-and-underestimates of factual knowledge. Due to differences in scales, factual and perceived knowledge scores were converted to standardized t-score values and then the t-score values were subtracted from one another to create the discrepancy scores. Discrepancy scores were grouped into three different categories: underestimation, no/minimal discrepancy and overestimation of factual knowledge. Underestimation (i.e., high factual knowledge and low perceived knowledge) was defined as a t-score difference >5 in the positive direction. Accurate perceptions (i.e., no/minimal discrepancy between factual and perceived knowledge scores) was defined as a t-score difference within 5 points in either the negative or positive direction of zero. Overestimation (i.e., low factual knowledge and high perceived knowledge) was defined as a t-score difference >5 in the negative direction. Independent samples t-tests, analysis of variance and chi-square tests were used to examine the relationship between parental biopsychosocial factors and parental knowledge and attitudes. Chi-square tests with odds risk ratios were used to examine parental biopsychosocial factors and discrepancy scores. Three linear regression models were used to examine associated child factors and parental knowledge and attitudes scores: model-1 factual knowledge, model-2 perceived knowledge and model-3 attitude score. To control for multiple children per parent, parent data was coded, and each parent was only included once in the analysis. In analyses that had both child and parent factors, each parent was only included once, and all children were included in child data analyses.

All data analyses were conducted with Statistical Package for the Social Sciences (SPSS) version 23 and 26 (IBM corp. Released, 2015 and 2019). Appropriate post-hoc analyses were conducted and effect sizes were reported for analyses. Bonferroni was used to control for multiple comparisons for each individual analysis, adjusting for the number of pairwise comparisons. All significant findings were evaluated for significance using the adjusted familywise error rate. For all analyses, two-tailed alpha values of less than 0.05 were considered statistically significant.

### Ethical considerations

The study was approved by the Human Participants Review Sub-committee of York University's Ethics Review Board in Toronto, Ontario.

## Results

Predominantly mothers completed the study questionnaire (73%). Parents were approximately 41 years old at the time of the study completion, and the average child age was 9.5 years. Eighty-nine percent of parents were Caucasian. Children were predominantly engaged in swimming (54%), soccer (39%), hockey (31%) and basketball (21%). There were six children that were not currently engaged in sport (See Table 1).

Parents scored an average of 76% accuracy on concussion factual knowledge (*M*_*average score*_ = 21.29, *SD* = 2.44). Incorrect sign/symptoms of an acute concussion most commonly selected by parents were difficulty speaking (62%), panic attacks (31%), and reduced breathing rate (25%; See Table 2). There were three statements that were frequently answered incorrectly: 49% of parents indicated that “*after a concussion occurs, brain imaging (e.g., CAT scan, MRI, x-Ray) typically show visible physical damage (e.g., bruise, bleed, blood clot)*”; 89% of parents indicated that “*an athlete who gets knocked out after getting a concussion is experiencing a coma*”; and 82% of parents reported that “*symptoms of a concussion typically last longer than 10 days*”, which were incorrect responses on the RoCKAS [34]. Please see Table 3.

**Table 2.** Factual knowledge: concussion signs and symptoms.

Sign or symptom (n = 78)	Response selected, n (%)
Headache^†^	69 (97%)
Difficulty speaking	44 (62%)
Arthritis	–
Sensitivity to light^†^	64 (90%)
Difficulty remembering^†^	62 (87%)
Panic attacks	22 (31%)
Drowsiness^†^	56 (79%)
Feeling in a “fog”^†^	64 (91%)
Weight gain	2 (3%)
Feeling slowed down^†^	50 (70%)
Reduced breathing rate	18 (25%)
Excessive studying	1 (1%)
Difficulty concentrating^†^	63 (89%)
Dizziness^†^	64 (90%)
Hair loss	–

† Indicates correct symptoms.

12 parents had missing data on this questionnaire and were excluded.

**Table 3.** Factual knowledge: statements about sustaining a concussion.

Actual knowledge (n = 78)	Correct responses, n (%)	Incorrect responses, n (%)
There is possible risk of death if a second concussion occurs before the first one has healed	61 (86%)	9 (13%)
Running everyday does little to improve cardiovascular health	64 (90%)	7 (10%)
People who have had one concussion are more likely to have another concussion	51 (72%)	20 (28%)
Cleats help athletes' feet grip the playing surface	67 (94%)	4 (6%)
In order to be diagnosed with a concussion, you have to be knocked out	69 (97%)	1 (1%)
A concussion can only occur if there is a direct hit to the head	67 (94%)	4 (6%)
Being knocked unconscious always causes permanent damage to the brain	62 (87%)	9 (13%)
Symptoms of a concussion can last for several weeks	70 (99%)	–
Sometimes a second concussion can help a person remember things that were forgotten after the first concussion	61 (86%)	8 (11%)
Weightlifting helps to tone and/or build muscle	68 (96%)	3 (4%)
After a concussion occurs, brain imaging (e.g., CAT scan, MRI, x-Ray) typically show visible physical damage (e.g., bruise, bleed, blood clot)	36 (51%)	34 (49%)
If you receive one concussion and you have never had a concussion before, you will become less intelligent	71 (100%)	–
After 10 days, symptoms of a concussion are usually completely gone	13 (18%)	58 (82%)
After a concussion, people can forget who they are and not recognize others but be perfect in every other way	43 (61%)	28 (39%)
High-school and college freshmen tend to be the same age	69 (97%)	2 (3%)
Concussions can sometimes lead to emotional disruptions	67 (94%)	2 (3%)
An athlete who gets knocked out after getting a concussion is experiencing a coma	8 (11%)	63(89%)
There is rarely a risk to long-term health and well-being from multiple concussions	66 (93%)	5 (7%)

There are some parents (n = 12) that did not complete every question and therefore, there are some missing data.

Parents on average reported feeling confident in their responses about knowledge symptoms and recovery (i.e., 74% confident in their responses; range 38–100%). There were 14 (16%) parents that had overestimated their factual knowledge (higher perceived knowledge than factual knowledge; scores ranged from -8.83 to 36.84). Thirty-six (40%) parents had accurate perceptions of their factual knowledge, and there were 26 (29%) parents that had underestimated their factual knowledge (higher factual knowledge than they perceived; scores ranged from 6.19 to 33.40). Parents, on average, displayed a generally favorable attitude (high positive) toward concussion reporting and management (*M* = 48.45, *SD* = 4.46, range 36–54).

Twenty-eight parents (31%) reported that they had sustained a concussion in the past, with the number of concussions ranging from 1 to 5 (See Table 1). Number of children, parental age at study completion, sex of the informant and parental concussion history were not associated with parental factual or perceived knowledge, discrepancy scores, or attitudes on concussion reporting and management (p = 0.15 to p = 0.96). There were differences in level of perceived knowledge based on parental SRS (F(2,72) = 6.34, p = 0.003), with parents with low SRS having higher perceived accuracy of knowledge than those with medium SRS (p = 0.019, d = 0.96), or high SRS (p = .035, d = 0.95). There was also a significant difference for SRS groups and discrepancy scores (χ^2^(4) = 9.80, p = 0.04, Cramer's V = .26). Factual knowledge scores and attitude scores were similar across SRS groups (See Table 4). Families with medium SRS were 15-times more likely to underestimate their factual knowledge compared with families with low SRS (95% CI: 1.68–133.92); and families with high SRS were 2.7-times more likely to underestimate their factual knowledge compared with families with low SRS (95% CI: 0.62–11.53). Furthermore, families with medium SRS were 5.5-times more likely to overestimate their factual knowledge about concussions compared with families with low SRS (95% CI: 0.45–66.31); yet families with low and high SRS had relatively equal odds of overestimating their factual knowledge about concussion (OR: 0.068, 95% CI: 00.07–6.78).

**Table 4.** Participant descriptives by social risk status.

Parent factors (n = 89)^†^	Low social risk, n (%) or mean (standard deviation)	Medium social risk, n (%) or mean (standard deviation)	High social risk, n (%) or mean (standard deviation)
	n = 62	n = 13	n = 14
Parent or caregiver participating			
Mother	44 (71%)	8 (62%)	13 (93%)
Father	15 (24%)	5 (38%)	1 (7%)
Both parents/caregiver	2 (3%)	–	–
Number of children in the family			
One child	27 (44%)	10 (77%)	10 (71%)
Two children	29 (47%)	2 (15%)	4 (29%)
Three children	6 (9%)	1 (8%)	–
Parent age	41 years 7 months (5 years 0 months)	41 years 5 months (6 years 10 months)	42 years 5 months (9 years 3 months)
Parents with a concussion history	17 (27%)	4 (31%)	7 (50%)
Factual knowledge score	21.36 (2.64)	21.27 (1.62)	21.10 (2.18)
Perceived knowledge score	16.31 (2.35)	13.90 (3.38)	14.10 (2.23)
Attitude score	48.24 (4.51)	48.55 (3.78)	49.00 (5.23)

Child factors (n = 140)	n = 108	n = 13	n = 17
Child age	9 years 3 months (2 years 8 months)	9 years 4 months (3 years 9 months)	10 years 6 months (3 years 1 months)
Child age when first began sports	3 years 9 months (1 year 2 months)	4 years 4 months (1 years 11 months)	3y 10m (1 year 5 months)
Children with a concussion history	17 (16%)	3 (23%)	3 (18%)
Child sport contact level			
Collision	41 (38%)	3 23%)	5 (29%)
Contact	29 (27%)	6 (46%)	7 (41%)
Limited/no contact	34 (32%)	4 (31%)	3 (18%)

† One parent did not complete questions on the social risk score variables.

Of the 140 children in the study, 16% had a history of concussion (number of concussions ranging from 1 to 3). Youth were relatively equally distributed among sport contact level with 37% engaging in collision level sports, 31% engaging in contact level sports and 31% engaging in limited or no contact level sports. Parental factual knowledge, perceived knowledge and attitude scores did not differ by the number of children in the home and therefore all children were included in the regression analyses. No child factors were significantly associated with discrepancies between parental perceived and factual knowledge (*p*s = .12-.63).

A series of linear regression models were conducted to examine child factors associated with parental factual knowledge, perceived knowledge and attitudes scores. The model examining factual knowledge was not significant (F(4,106) = 1.53, p = 0.20, *R*^2^ = 0.06). However, age at which the children began playing sports was significant (β = -0.22, p = 0.03), with the younger age at which the children begin sports being correlated with higher factual knowledge. The model examining parental attitude scores and child factors was also not significant (F(4,105) = 1.20, p = 0.32, *R*^2^ = 0.04). Although not significant (β = -0.19, p = 0.056), post-hoc analysis revealed that parents of children with a concussion history had a more favorable attitude about concussion reporting and management than parents of children without a concussion history (t(117) = 20.05, p = 0.04, d = 0.49). The linear regression model for perceived knowledge was also not significant (F(4,104) = 2.37, p = 0.057; *R*^2^ = 0.084); however, current level of sport contact significantly predicted parental perceived knowledge scores (β = 0.27, p = 0.006). Parents with children participating in collision and contact level sports had similar perceived knowledge of concussions (p = 0.38). However, those with children in collision level sports had higher perceived knowledge of concussions compared with parents with children in the limited/no contact sport level (p = 0.037, d = 0.70). Parents that had children in contact sport level had similar perceived knowledge than parents of children in limited/no contact sport level (p = 0.96).

## Discussion

Parents demonstrated a moderate amount knowledge about concussion signs and symptoms, similar to rates found in prior literature including Public Health Agency of Canada (PHAC) [32], and other studies conducted in USA that used mixed samples [13,17,18,32,36]. A substantial proportion of parents incorrectly identify reduced breathing rate, difficulty speaking and panic attacks as signs/symptoms of an acute concussion and reported that they believed physical damage to the brain following a concussive injury can be observed on clinical brain scans (e.g., MRI, CT) [32]. These findings identify potential targets for education efforts to improve knowledge of concussion signs and symptoms for parents.

Despite it being an incorrect answer on the RoCKAS, 82% of parents agreed with the statement that concussion recovery typically takes longer than 10 days which is aligned with current literature and concussion education platforms. For example, education platform such as CDC-HEADS UP program and Parachute Canada provide more broad guidelines for concussion recovery (i.e., taking up to a couple of weeks) [37,38]. Emerging research also suggests longer recovery times for youth and high-school age children (~15–20 days) [39,40], with even longer recovery times for cognitive symptoms [11,41,42]. Our research, thus, highlights the importance of disseminating consistent and accurate information about recovery trajectories that may vary across age, sex and various athlete and non-athlete populations.

In the current study, parents had similar factual knowledge scores about concussion signs/symptoms yet, families with higher SRS perceived themselves to have lower factual knowledge compared with families with lower SRS despite relative homogeneity of social status in the sample. Prior research demonstrates individual indicators of social status such as household income, parental education and stressors associated with lower SES (i.e., higher SRS) has a negative impact on parental attitudes about concussions and confidence in general [13,20,29,30]. Together these findings suggest that SRS may not be associated with actual knowledge rather there may be an opportunity to increase parental confidence and perceived knowledge among higher risk families within the communities in the current study. The current study highlights a need to explore types of psychoeducational approaches among various community samples, as well as other methods that would improve confidence among parents about concussion knowledge.

Parents who enrolled their children in sports at a younger age were more knowledgeable about concussions than parents who enrolled their children at an older age. It is possible that parents who have a child in sports for a longer period of time are more likely to seek out concussion information as it is directly relevant to their child's sport participation, and they are more likely to be exposed to concussion information through sport organizations [43,44]. Additionally, higher perceived knowledge was associated with parents with children in contact sports, and a more favorable attitude about concussion reporting and management was associated with a positive child concussion history. A positive attitude about concussion reporting and management is likely endorsed by parents of children with a history of concussion because they have utilized concussion management strategies in the past [44]. Parents who have children engaged in higher contact level sports receive more formal or informal information about concussions than parents of children within the limited or no contact sport groups. Importantly, more than half the parents in the Canadian PHAC baseline study believed that averting engagement in contact sports will prevent concussions [39]. It is important to ensure that concussion knowledge is disseminated equally across all levels of contact in order to provide parents with the tools and confidence to make informed decisions about sport participation and preserve engagement in youth sports of all levels.

There are additional avenues that that could not be addressed by the current study due to practical and other limitations. The study's participants were from a small sample of parents recruited from the GTA that are likely active in the community and/or have youth involved in sports, thus limiting the diversity and generalizability of the findings. Furthermore, the rate of refusal was unable to be accurately tracked and therefore was not able to be analyzed. Ninety-six percent of our sample of children were enrolled in sports which exceeds the national average of approximately 75% in Canada [45], despite our attempt to mitigate this issue and capture a more diverse sample by including public libraries, and community/recreational centers. Despite using a multifaceted robust measure of social risk, our sample was fairly homogeneous in nature. Nevertheless, we were able to find notable differences between social risk groups and perceived knowledge. However, the homogeneity of the groups poses a potential bias and future research is warranted to further understand these relationships within more diverse populations. Future research is also warranted to better understand how child sex can influence parent knowledge of concussions and attitudes toward concussion reporting and management, given our inability to examine this due to a lack of data related to youth sex.

## Conclusion

Despite these limitations, the results from this study compliment the 2017 Berlin Consensus Statement on Concussion in Sport [41], and offer a breadth of information that may be useful for informing knowledge translation platforms and education initiatives in our community, adding to the pan-Canada goal of raising awareness for parents, coaches and athletes about concussion knowledge, recovery and treatment by the Ministry of Health and Ministry of Sport and Persons with Disabilities. This study offers unique insight into parental knowledge and attitudes of sport-related concussion in a small subset of Canadian parents (i.e., from the GTA). It identifies factors that encumber parental confidence, knowledge, and attitudes about concussion that may lead to a reduction in sport participation. This study also helps inform knowledge translation platforms and policies to increase parental factual and perceived knowledge about concussion, supporting youth sport engagement. Specifically, we can target misconceptions about concussion among all families within Ontario by providing accurate and up-to-date information about concussions uniformly across parents. Furthermore, we can aim to increase parental perceived knowledge about concussions among families that experience high social risk factors. It is important to tailor education initiatives to increase knowledge, confidence and attitudes of parents due to the influence this has on youth flourishing socially and developmentally in community sports. There is variability about concussion management among health sectors in Canada [32], and this study helps to fill the gap by highlighting signs and symptoms that parents incorrectly identify, to support the national goal of creating a comprehensive pan-Canadian approach to concussion prevention, detection and management. Collectively, the results from the current study can be used as a stepping stone to inform future research and government bodies as they improve the quality, format and scope of concussion education through new initiatives or existing platforms, in order to promote active youth and their participation in sport.

**Executive summary**

- The current cross-sectional study aimed to better understand parental knowledge and attitudes regarding paediatric sport-related concussions, and associations with parent and child biopsychosocial factors.
- Parents scored an average of 76% accuracy on factual concussion knowledge, with 74% confidence in their responses.
- Parents endorsed a favourable attitude towards concussion reporting and management.
- Low SRS had higher perceived accuracy of knowledge than medium or high SRS. SRS influenced over-and-underestimations of factual knowledge.
- This study offers a breadth of information that may be useful for informing knowledge translation platforms and education initiatives in our community, adding to the pan-Canada goal of raising awareness for parents, coaches, and athletes about concussion knowledge, recovery, and treatment by the Ministry of Health and Ministry of Sport and Persons with Disabilities.
- It helps identify factors that encumber parental confidence, knowledge, and attitudes about concussion that may lead to a reduction in sport participation. We can aim to increase parental perceived knowledge about concussions among families that experience high social risk factors. It is important to tailor education initiatives to increase knowledge, confidence, and attitudes of parents due to the influence this has on youth flourishing socially and developmentally in community sports.
- This study also helps inform knowledge translation platforms and policies to increase parental factual and perceived knowledge about concussion, supporting youth sport engagement. Specifically, we can target misconceptions about traumatic brain injury and concussion among all families within Ontario by providing accurate and up-to-date information about concussions uniformly across parents.
- This study helps to fill the gap by highlighting signs and symptoms that parents incorrectly identify, in order to support the national goal of creating a comprehensive pan-Canadian approach to concussion prevention, detection, and management.
- Collectively, the results from the current study can be used as a steppingstone to inform future research and government bodies as they improve the quality, format, and scope of concussion education through new initiatives or existing platforms, to promote active youth and their participation in sport.
